# A chaos-based augmented image encryption scheme for satellite images using Fredkin logic

**DOI:** 10.1038/s41598-025-22008-z

**Published:** 2025-10-27

**Authors:** Wassim Alexan, Engy Aly Maher, Eyad Mamdouh, Mohamed Youssef, Noha Ehab

**Affiliations:** 1 Communications Department, Faculty of Information Engineering and Technology, German University in Cairo, Cairo, 11835 Egypt; 2 Computer Science Department, Faculty of Media Engineering and Technology, German University in Cairo, Cairo, 11835 Egypt; 3 Networking Department, Faculty of Information Engineering and Technology, German University in Cairo, Cairo, 11835 Egypt

**Keywords:** Chaos theory, Fredkin, Gauss circle map, Hill cipher, Image encryption, Hyperchaos, S-box, Electrical and electronic engineering, Applied mathematics, Computer science, Information technology

## Abstract

This article presents a novel augmented image encryption algorithm tailored for securing satellite images, addressing the critical need for robust protection of sensitive geographic data. Implementing Shannon’s principles of confusion and diffusion, the method begins by augmenting multiple plain images into a single large image, followed by a three-stage encryption process. Initially, the augmented image is separated into its three color channels, which are transformed into one-dimensional (1D) bit-streams, split and altered using the Gauss Circle map, and restructured via Fredkin Gates to enhance unpredictability. Subsequently, the bit-streams are converted into 1D bytes and $2 \times 2$ matrices, processed through three systems incorporating hyperchaos-induced keys and dynamic Hill Cipher matrices for additional confusion and diffusion. The final stage combines these encrypted streams into one image while preserving the integrity of color data. The proposed method achieves strong security metrics, including an average Number of Pixels Change Rate (NPCR) of $99.6115\%$, a Unified Average Changing Intensity (UACI) of $31.71\%$, and high entropy values (e.g., 7.9989) for encrypted images, ensuring robust resistance to differential and statistical attacks. The encryption demonstrates computational efficiency with an encryption time of 0.2817s for $256\times 256$ images and maintains a low Peak Signal-to-Noise Ratio (PSNR) of 8.1 dB, reflecting effective data obfuscation. This multistage chaos-based approach, leveraging Fredkin logic gates and hyperchaos-induced keys, significantly enhances security, scalability, and efficiency, making it ideal for high-stakes satellite imagery applications where data integrity and confidentiality are paramount.

## Introduction

In the field of satellite imaging, where the acquisition and transmission of high-resolution images play critical roles in diverse sectors such as national security, environmental monitoring, and urban planning, the safeguarding of this sensitive data is of utmost importance^1^. Traditional encryption algorithms such as the Data Encryption Standard (DES), Advanced Encryption Standard (AES), and International Data Encryption Algorithm (IDEA) have been instrumental in protecting data. However, these methods often do not fully meet the unique demands of satellite imagery, where both high security and minimal loss of image detail are required^2^. Satellite images can contain sensitive information ranging from strategic military installations to critical infrastructure details, making their security a top priority^3^. Satellite images differ from regular images in several critical aspects that influence encryption requirements. First, satellite images typically possess higher resolutions and larger file sizes, which necessitate encryption algorithms that are both computationally efficient and scalable^4^. Second, satellite data often contains sensitive geographic and strategic information (e.g., military installations, environmental monitoring zones), which raises the bar for security against statistical and differential attacks^5^. Third, satellite imagery is frequently used in real-time or near-real-time applications, such as disaster management and surveillance, demanding low-latency encryption and decryption^6^. Lastly, satellite images may undergo various pre-processing steps like radiometric correction and georeferencing, which require that the encryption process preserves data integrity and format compatibility. These requirements are more stringent than those for regular multimedia images, where minor losses and delays are more tolerable.

Unlike traditional approaches that encrypt each image individually in sequence, multiple image encryption schemes combine several images into a single augmented representation before encryption^7,8^. This strategy not only reduces the overall encryption time by enabling parallelized or batch processing, but also enhances security by increasing data complexity and entropy^9^. It is particularly beneficial in big data and remote sensing contexts, where handling multiple high-resolution satellite images simultaneously is common^10^.

In the field of multimedia security, the application of chaos theory has become particularly prominent, offering a robust framework for the encryption of diverse content forms^11^. Utilizing the inherent unpredictability and sensitivity to initial conditions characteristic of chaotic systems, chaos-based encryption methods introduce a high degree of non-linearity and complexity^12–14^. This is crucial for thwarting a variety of cryptographic attacks, enhancing the security of multimedia files. Specifically, in image encryption, these chaotic properties facilitate the generation of pseudo-random sequences that are used to permute and diffuse pixel values effectively, thus obscuring original image content^15,16^. The iterative nature of chaotic maps allows for dynamic and adjustable security layers, significantly enhancing protection. Additionally, chaos-based image encryption algorithms are known for their efficiency, making them ideal for real-time applications and suitable for environments with constrained computational resources^17,18^. This adaptability does not compromise the file size or format, which is essential for large-scale applications such as satellite imagery or streaming services^1,7^. Overall, the integration of chaos theory into multimedia encryption ensures robust security, maintaining both the integrity and confidentiality of digital media^19^.

The use of logical operators such as XOR (Exclusive OR)^20^, XNOR (Exclusive NOR)^21^, the Fredkin gate^22^, and the CNOT (Controlled NOT) gate^23^, plays a crucial role in the field of data encryption, particularly for multimedia content like satellite images. The reversible property of these gates, where the input can be derived from the output without any loss of information, is particularly appealing in multimedia encryption. This reversibility ensures that the original high-quality images can be perfectly reconstructed after decryption, an essential factor for applications where detail and accuracy are paramount^21,24^. The XOR and XNOR operators are favored in image encryption algorithms due to their simplicity and effectiveness in altering the image data to secure it against unauthorized access. The Fredkin gate and CNOT gate offer additional security benefits; the Fredkin gate performs controlled swaps based on the input condition, and the CNOT gate provides a bitwise conditional operation that is fundamental in quantum computing and adds a layer of complexity and robustness to the encryption process, enhancing its resistance to cryptographic attacks^25^.

This article significantly advances the field of multimedia encryption with several key contributions:

1. Chaos theory application: It introduces a novel chaos-based image encryption scheme tailored specifically for satellite images, enhancing security through increased unpredictability.
2. Augmented image encryption: The method combines multiple plain images into one before encryption, increasing process efficiency and security complexity.
3. Three-stage encryption process: It details a sophisticated method involving transformation, manipulation using the Gauss Circle map, and restructuring via Fredkin gates, adding depth to the security measures.
4. Hyperchaos keys and dynamic Hill cipher: The integration of these elements introduces additional layers of security, dynamically adapting to ensure consistent protection.
5. Security and efficiency evaluation: The article provides a comprehensive analysis of the method’s security against various attacks (i.e. a key space of $2^{4464}$) and demonstrates its practicality for real-time applications (i.e. an encryption rate of 5.98 Mbps, essential for handling large-scale satellite image data.
6. Overall, the contributions of this article improve the encryption of satellite images employing advanced cryptographic techniques and thorough security evaluations.

The structure of this article is as follows: Section "Related literature" discusses recent related literature. Section "Foundational mathematical constructs" outlines the foundational mathematical constructs. Section "Proposed algorithm" details the encryption and decryption processes. Section "Performance evaluation" showcases the outcomes of implementing the proposed cipher on different examples and evaluates its effectiveness through visual, statistical, and quantitative measures. Finally, Section "Conclusions and Future Works" provides the conclusions of this article and suggests possible future research directions that may be further pursued.

## Related literature

The evolution of image encryption has witnessed remarkable developments over the past decade, driven by growing concerns about data security in various domains, including geographic information systems (GIS). While traditional cryptographic methods struggled with the unique challenges posed by large-scale satellite imagery, recent advances in chaos theory and quantum-inspired algorithms have opened new pathways for secure image transmission. This section examines key developments in image encryption techniques, focusing particularly on approaches that leverage multiple encryption stages and dynamic key generation. Special attention is given to methods incorporating Shannon’s confusion-diffusion principles^26^ and their applications in securing sensitive geographic data^1^. The following paragraphs attempt to portray the state of the current image encryption literature.

### Chaos-based image encryption methods

Chaos-based methods have gained significant traction due to their inherent properties of unpredictability, sensitivity to initial conditions, and ergodicity. In^20^, the authors present an image encryption algorithm combining unique image transformations with chaotic and hyper-chaotic systems, leveraging the Chua and Chen systems for rescaling, rotation, and randomization. Their approach offers a vast key space of $2^{5208}$, ensuring strong security. The work in^21^ introduces an encryption method using base-*n* Pseudo-Random Number Generators (PRNGs) and parallel base-*n* S-boxes, hybridizing chaotic systems (e.g., Chen and Chua) with memristor circuits. This method enhances resistance to attacks while achieving parallelization for faster processing. The authors of^27^ proposed a chaos-based encryption scheme using random chaos sequences for pixel permutation and diffusion. Sensitivity to initial conditions and control parameters ensures high security. In^28^, the authors introduced a hybrid scheme combining the Lorenz chaotic system with an inverse left almost semigroup structure. This multi-stage confusion-diffusion process demonstrated strong robustness against differential and linear attacks, making it suitable for multimedia security.

### Chaotic map combinations for efficiency and security

Several works have explored the combination of chaotic maps to achieve simplicity, efficiency, and high-security margins. The combined use of Hénon and logistic chaotic maps was proposed in^29^. This permutation- and diffusion-based algorithm achieves high sensitivity to initial conditions, ensuring robustness against statistical and differential attacks. The authors of^30^ propose a confusion-diffusion encryption technique where image pixels are shuffled and then diffused by XORing with a secret key generated from multiple chaotic maps. Specifically, the Arnold cat map, the 2D logistic sine map, the linear congruential generator, the Bernoulli map, and the tent map. The work in^31^ employs multiple chaotic iterative maps, including the Hénon, Duffing, and circle chaotic maps, alongside the Rand block function. The confusion-diffusion mechanism enhances encryption strength and ensures secrecy.

### S-box construction for cryptographic applications

S-boxes play a critical role in block ciphers and image encryption by introducing non-linearity and resistance to various attacks. In^32^, the authors propose constructing S-boxes using points on an elliptic curve over a prime field. These S-boxes are evaluated for resistance to linear, differential, and algebraic attacks, demonstrating strong cryptographic properties. The authors of^33^ introduced a method for constructing S-boxes using Gaussian distribution and linear fractional transform. The design employs the Box-Muller transform and central limit algorithm, achieving high cryptographic strength based on standardized tests. A secure image encryption scheme combining an algebraic S-box with the scrambling effect of the Arnold transform was presented in^34^. The algebraic structure of the multiplicative cyclic group of the Galois field enhances both security and computational efficiency. The work in^35^ proposes constructing S-boxes using cubic fractional transformation (CFT). Evaluated against criteria such as bijection, non-linearity, and avalanche effect, this method demonstrates strong potential for generating efficient S-boxes for block ciphers.

### Multi-layer encryption, FPGA implementations and UAV-assisted networks

Recent advancements focus on multi-layer encryption schemes and secure transmission protocols for sensitive applications. To increase encryption efficiency, the authors of^36^ present an image encryption technique leveraging three hyperchaotic systems: a memristor system, a hyperchaotic 7D system, and an Erbium-doped fiber laser system, for a robust three-layer encryption process. Using PRNGs, it constructs S-boxes and encryption keys to secure image data. Implemented in software (Wolfram Mathematica^®^ 13.1) and hardware (AMD Virtex-7 FPGA VC707), the scheme achieves encryption rates of 9.7 Mbps and 0.67 Gbps, respectively. In^37^, the authors propose a 2-layer image encryption cryptosystem for transmitting military reconnaissance images over UAV-assisted networks. The first layer employs a Genetic Algorithm (GA) with a Mersenne Twister (MT) key, followed by DNA coding for added security. The encrypted images are channel-coded (a convolutional or an LDPC code) and transmitted via multi-hop UAV relays, ensuring robust security and efficiency in hostile environments.

### Summary and research gap

The reviewed literature highlights significant advancements in chaos-based image encryption, including multi-stage encryption, dynamic key generation, and the integration of chaotic maps and S-boxes to enhance security and efficiency. While these methods demonstrate robustness against various cryptographic attacks, they often lack a focus on augmenting multiple images into a single encryption process, which is critical for compressing and securing large-scale satellite data. Furthermore, the potential of quantum-inspired logic gates, such as Fredkin gates, remains largely unexplored in this domain. This research addresses these gaps by proposing a novel encryption scheme that combines image augmentation, chaos theory, and Fredkin logic to achieve enhanced security and scalability for satellite imagery applications.

## Foundational mathematical constructs

### Fredkin gate

**Fig. 1:**
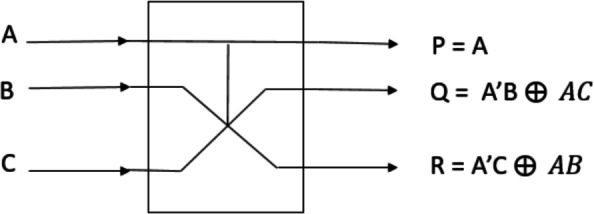
An illustration of the Fredkin gate.

Fredkin gate is one of the most flexible and reversible gate types. As shown in Fig. 1, the Fredkin gate takes a 3-bit input; each bit labelled as *A*, *B* and *C*, and generates a 3-bit output; each bit labelled as *P*, *Q* and *R* after processing. The output bit *P* in this gate has the same value as the input bit *A*. Based on the value of bit *A*, if it is zero, then *Q* will be equal to *B* and *R* will be equal to *C*. However, when *A* is one, *Q* will be equal to *C* and *R* will be equal to *B*. The values of *Q* and *R* are deduced from the value of *A*. In simple terms, the Fredkin gate only switches *B* and *C* when *A* is 1, keeping the value of *A* unchanged. It is referred to as the controlling swap gate, or CSWAP, because of the way it functions. The Truth table of the Fredkin gate is shown in Table 1.

**Table 1:** Truth table of the Fredkin gate.

**Inputs**			**Outputs**		
A	B	C	P	Q	R
0	0	0	0	0	0
0	0	1	0	0	1
0	0	1	0	0	1
0	1	0	0	1	0
0	1	1	0	1	1
1	0	0	1	0	0
1	0	1	1	1	0
1	1	0	1	0	1
1	1	1	1	1	1

### Gauss circle chaotic map

In^38^ the authors construct the Gauss Circle map. This map is a recursive function that integrates the Gauss map^39^, and the circle map^40^. From^38^ the equation of the Gauss Circle map is:

1 $$\begin{aligned} x_{i+1}=e^{-\alpha (\frac{5}{4} \times ((x_i+\Omega +\frac{\lambda }{2\times \pi }\sin {(2\pi x_i)}))\bmod {1})-\frac{1}{2})^2}+\beta , \end{aligned}$$

where $\alpha =9$, $\beta =0.481$, $\lambda =10^6$, $\Omega =0.5$ and $x\in (0,1)$ are the best choices for a chaotic behaviour^38^, iterations of an initial value $x_0$ result in chaotic rather than periodic trajectories over the interval $(0,1)$, due to the high precision of the system’s parameters. Permutation is achieved through the generation of highly chaotic sequences using Gauss Circle maps. The Gauss Circle map sequence successfully passed all 16 NIST tests and demonstrated a robust defense against entropy attacks^38^.

### 6D hyperchaotic system

The authors of^41^ construct a six-dimensional (6D) hyperchaotic system. The system is defined by the following set of differential equations:

2 $$\begin{aligned} \begin{aligned} \left\{ \begin{aligned}&\dot{x}_1=\beta _1(x_2-x_1)+x_4,\\&\dot{x}_2=-\beta _2 x_2 - x_1 x_3 + x_6,\\&\dot{x}_3=-\beta _3 + x_1 x_2,\\&\dot{x}_4= -x_2 + x_5,\\&\dot{x}_5= \beta _4 x_2 +x_4,\\&\dot{x}_6= \beta _5 x_1 + \beta _6 x_2,\\ \end{aligned} \right. \end{aligned} \end{aligned}$$

where $X=(x_1,x_2,x_3,x_4,x_5,x_6)$ represents the states of the hyperchaotic system, while $(\beta _1,\beta _2,\beta _3,\beta _4,\beta _5,\beta _6)$ are parameters that govern the chaotic behavior of the system. The system exhibits hyperchaotic behavior when $\beta _1 \in [15, 4.273]$, $\beta _2=2.7$, $\beta _3=-3$, $\beta _4=2$, $\beta _5=100$, and $\beta _6=1$. The chaotic behavior is verified through the calculation of the Lyapunov exponents, which are given as $\lambda _1=1.3613$, $\lambda _2= 0.0733$, $\lambda _3= 0.0478$, $\lambda _4=0.0189$, $\lambda _5=0$ and $\lambda _6=-14.201$. Since there are four positive Lyapunov exponents, this system is classified as hyperchaotic.

Additionally, the authors identified singularly degenerate heteroclinic cycles and bifurcations leading to hidden hyperchaotic attractors through analytical methods and numerical simulations. The dynamics have been confirmed through the 0-1 test and circuit testing^41^.

### 8D hyperchaotic system

The authors of^42^ analyzed the dynamic properties of a new 8D hyperchaotic system. The system is defined by the following set of differential equations:

3 $$\begin{aligned} \begin{aligned} \left\{ \begin{aligned}&\dot{x}_1=\beta _1 (x_2-x_1) +x_4,\\&\dot{x}_2=-\beta _2 x_1 - x_1 x_3 + x_4,\\&\dot{x}_3=-x_1 x_2 + x_3 - x_4 + x_7,\\&\dot{x}_4= -\beta _3 (x_1+x_2) + x_5,\\&\dot{x}_5= -x_2 - \beta _4 x_4 + x_6,\\&\dot{x}_6= -\beta _5 (x_1+x_5) + \beta _4 x_7,\\&\dot{x}_7= -\beta _6 (x_1 + x_6 - x_8),\\&\dot{x}_8= -\beta _7 x_7,\\ \end{aligned} \right. \end{aligned} \end{aligned}$$

where $X=(x_1,x_2,x_3,x_4,x_5,x_6,x_7,x_8)$ indicates the 8D hyperchaotic system’s variables, $(\beta _1,\beta _2,\beta _3,\beta _4,\beta _5,\beta _6,\beta _7)$ are parameters that determine the chaotic behavior of the system when the initial values of the variables are set as $X=(1, 1, 1, 1, 0, 0, 0, 0)$, $\beta _1=10$, $\beta _2=76$, $\beta _3=3$, $\beta _4=0.2$, $\beta _5=0.1$, $\beta _6=0.1$ and $\beta _7=0.2$. The characteristic Lyapunov exponents are computed as $\lambda _1=1.4565$, $\lambda _2=0.1176$, $\lambda _3=0.0623$, $\lambda _4=0.0433$, $\lambda _5=0.0260$, $\lambda _6= 0.0132$, $\lambda _7=0$ and $\lambda _8 =-12.6987$. Since there are 6 positive Lyapunov exponents, this system shows a good hyperchaotic behavior according to^43^. To evaluate the system’s physical feasibility, an equivalent electronic circuit was created using the Multisim^®^ software in^42^. Using an 8D hyperchaotic system with 23 terms and 7 control parameters, the suggested algorithm produces a large key space and an extremely safe encryption technique that has a low time complexity.

### Hill cipher

The Hill cipher, introduced by Lester S. Hill in 1929, marked a significant milestone in classical cryptography. Unlike traditional substitution ciphers that operate on individual characters, the Hill cipher utilizes linear algebra to encrypt entire blocks of text simultaneously through matrix operations^44^. By leveraging the mathematical properties of matrices, it became one of the earliest polygraphic ciphers, where each ciphertext character is influenced by multiple plaintext characters. The decryption process, which depends on matrix inversion, combines cryptographic robustness with mathematical sophistication. Its application in modern cryptographic analysis not only emphasizes its historical significance but also demonstrates the continued relevance of algebraic methods in fields like image encryption^45^.

In the Hill cipher, plaintext blocks are represented as vectors, which are transformed into ciphertext vectors through multiplication with an encryption matrix $B$, followed by taking the result modulo $m$, where $m$ denotes the alphabet size. Let $\textbf{X}$ represent the plaintext vector and $\textbf{Y}$ the ciphertext vector. The encryption process can be expressed as:

4 $$\begin{aligned} \textbf{Y} = B \cdot \textbf{X} \bmod m. \end{aligned}$$

Decryption reverses this process using the inverse of the encryption matrix, denoted as $B^{-1}$, provided it exists modulo $m$. This is possible only if the determinant of $B$ satisfies $\text {gcd}(\text {det}(B), m) = 1$. The decryption process is expressed as:

5 $$\begin{aligned} \textbf{X} = B^{-1} \cdot \textbf{Y} \bmod m. \end{aligned}$$

For both encryption and decryption, the matrices $B$ and $B^{-1}$ must have dimensions compatible with the length of the plaintext vector $\textbf{X}$. The parameter $m$ typically denotes the size of the alphabet (e.g., $26$ for the English alphabet). This configuration ensures that each letter (or block of letters, depending on the matrix size) from the plaintext is methodically converted into ciphertext, utilizing matrix operations to achieve cryptographic transformation.

## Proposed algorithm

### Encryption process

In this section, the construction of the encryption process is explained in the following steps.

1. Stage 1:

- Multiple plain images are generated and then formed into a single augmented image $I_{A}$.
- Separate the augmented plain image into 3 color channels; Red, Green and Blue and convert them into 1*D* bit-streams giving $I_{[A,1D,Red]}$, $I_{[A,1D,Green]}$ and $I_{[A,1D,Blue]}$.
- Given $Seed_{Gauss}$, 3 different bit-streams are generated from the Gauss circle chaotic map from (1) giving $Key_{GaussRed}$, $Key_{GaussGreen}$ and $Key_{GaussBlue}$.
- $I_{[A,1D,Red]}$ is divided into halves to be inputs of ***B*** and ***C*** for the Fredkin gate with $Key_{GaussRed}$ as input ***A*** giving $I_{[A,1D,Red,Fredkin]}$.
- $I_{[A,1D,Green]}$ is divided into halves to be inputs ***B*** and ***C*** for the Fredkin gate with $Key_{GaussGreen}$ as input ***A*** giving $I_{[A,1D,Green,Fredkin]}$.
- $I_{[A,1D,Blue]}$ is divided into halves to be inputs ***B*** and ***C*** for the Fredkin gate with $Key_{GaussBlue}$ as input ***A*** giving $I_{[A,1D,Blue,Fredkin]}$.

2. Stage 2:

- Given $Seed_{6D}$, 3 different S-boxes are constructed from the 6D hyperchaotic system in (2) giving $Sbox_{6Dred}$, $Sbox_{6Dgreen}$ and $Sbox_{6Dblue}$, as shown in Tables 2, 3 and 4.
- $I_{[A,1D,Red,Fredkin]}$ is converted into bytes and invoked with $Sbox_{6Dred}$ giving:
  6 $$\begin{aligned} I_{[A,1D,Red,Fredkin,Sbox]}=Sbox\big (I_{[A,1D,Red,Fredkin]}\big ) \end{aligned}$$
- $I_{[A,1D,Green,Fredkin]}$ is converted into bytes and invoked with $Sbox_{6Dgreen}$ giving:
  7 $$\begin{aligned} I_{[A,1D,Green,Fredkin,Sbox]}=Sbox\big (I_{[A,1D,Green,Fredkin]}\big ) \end{aligned}$$
- $I_{[A,1D,Blue,Fredkin]}$ is converted into bytes and invoked with $Sbox_{6Dblue}$ giving:
  8 $$\begin{aligned} I_{[A,1D,Blue,Fredkin,Sbox]}=Sbox\big (I_{[A,1D,Blue,Fredkin]}\big ) \end{aligned}$$

3. Stage 3:

- Given $Seed_{8D}$, 3 different $2\times 2$ Hill cipher matrices are generated from (3) giving $Key_{HillCipherRed}$, $Key_{HillCipherGreen}$ and $Key_{HillCipherBlue}$.
- $I_{[A,1D,Red,Fredkin,Sbox]}$ is converted into $2\times 2$ matrices then matrix multiplication is applied with $Key_{HillCipherRed}$ giving $I_{[A,Red,Fredkin,Sbox,2\times 2,Hill]}$.
- $I_{[A,1D,Green,Fredkin,Sbox]}$ is converted into $2\times 2$ matrices then matrix multiplication is applied with $Key_{HillCipherGreen}$ giving $I_{[A,Green,Fredkin,Sbox,2\times 2,Hill]}$.
- $I_{[A,1D,Blue,Fredkin,Sbox]}$ is converted into $2\times 2$ matrices then matrix multiplication is applied with $Key_{HillCipherBlue}$ giving $I_{[A,Blue,Fredkin,Sbox,2\times 2,Hill]}$.
- Finally, combine the 3 color channels together and reshape into an image. This produces an encrypted augmented image $I'_{A}$.

**Fig. 2:**
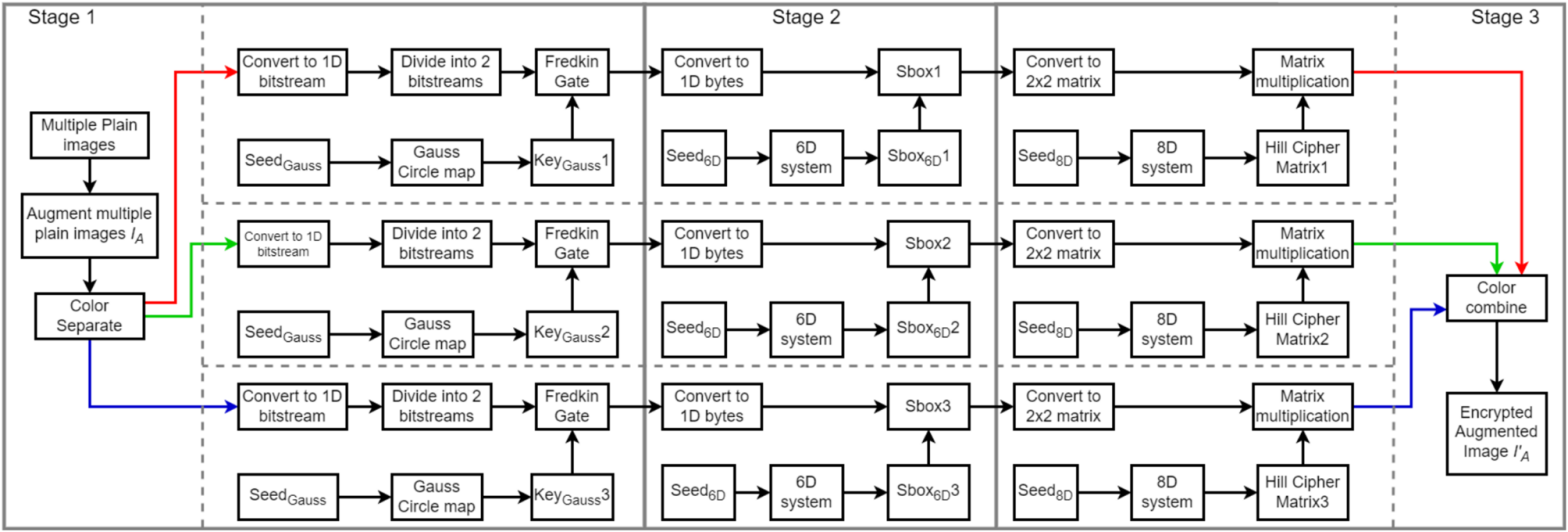
Encryption process flow chart.

An illustration of the encryption process is depicted in Fig. 2.

**Table 2:** S-box generated from the 6D hyperchaotic sequence specified for the Red image channel.

4	131	126	185	127	0	253	111	222	151	162	209	93	112	169	145
31	19	135	150	140	43	238	107	108	30	75	20	11	227	99	208
118	254	36	28	182	117	48	80	6	47	121	45	109	165	85	184
10	204	87	24	94	64	92	159	50	232	132	76	134	55	205	198
101	13	54	152	171	83	183	73	138	175	106	60	14	215	49	9
59	212	187	224	15	235	201	113	177	190	176	143	137	236	180	246
207	39	97	103	44	161	67	110	124	155	71	65	188	195	18	16
88	142	233	69	199	41	194	89	3	78	249	170	197	147	51	156
77	158	7	84	123	17	56	230	251	86	229	52	174	206	211	247
81	90	8	166	74	120	144	68	242	245	191	66	82	42	210	34
63	231	22	38	12	217	130	216	139	23	40	157	37	128	192	203
114	72	218	225	104	1	146	79	105	5	248	27	250	200	244	35
154	33	122	255	46	189	173	57	100	181	116	219	214	2	119	179
220	98	252	168	141	21	136	53	96	25	149	153	240	58	228	239
243	221	223	167	186	202	196	226	102	115	95	125	70	29	241	32
129	237	193	61	91	148	213	26	172	163	62	234	133	178	160	164

**Table 3:** S-box generated from the 6D hyperchaotic sequence specified for the Green image channel.

32	154	253	99	110	70	221	252	71	188	39	17	163	201	98	100
9	114	42	11	104	180	239	44	236	109	222	21	7	165	14	36
177	35	191	248	130	250	152	218	24	45	55	134	23	194	187	123
217	52	193	247	22	29	159	57	49	62	135	210	96	212	34	54
10	102	175	48	189	20	133	56	65	4	67	179	185	108	170	213
226	107	140	73	230	53	115	196	58	120	59	118	148	95	223	97
225	33	150	242	228	246	122	208	76	169	78	131	162	81	132	151
16	198	72	174	129	31	241	209	38	220	231	233	144	83	141	249
145	77	0	6	192	203	195	200	69	84	173	155	215	3	12	61
237	216	46	25	75	227	93	63	66	40	235	64	68	5	184	82
158	86	101	245	106	112	2	41	182	190	74	164	186	168	85	113
79	94	47	243	111	87	251	125	199	119	30	178	80	183	128	137
116	50	124	153	43	1	15	207	91	121	176	166	147	240	37	138
214	206	211	139	143	205	27	117	232	51	197	19	224	8	105	88
90	254	238	92	28	18	103	126	26	127	181	136	244	157	142	219
156	160	161	171	202	229	172	255	167	204	13	234	89	146	149	60

**Table 4:** S-box generated from the 6D hyperchaotic sequence specified for the Blue image channel.

93	72	23	156	132	183	101	207	170	106	224	70	69	96	6	126
136	74	97	73	29	202	37	40	18	246	20	250	117	128	127	41
4	43	164	129	3	158	116	161	28	140	119	5	45	152	36	133
197	51	38	199	146	216	138	123	90	163	30	239	35	55	242	244
80	99	60	94	186	188	27	155	109	25	105	212	13	59	120	234
54	115	160	195	135	125	154	46	147	39	26	175	100	24	245	66
12	174	11	253	159	76	53	210	81	137	173	194	178	139	232	14
16	209	78	82	84	165	221	86	228	243	229	176	32	254	220	206
196	200	87	9	203	230	8	83	31	222	47	21	33	7	68	237
75	56	22	0	225	172	79	113	44	122	88	98	85	249	168	71
61	204	110	118	65	192	121	144	62	17	218	217	104	58	211	89
50	49	103	114	241	201	102	108	15	34	57	189	179	130	213	167
67	142	2	42	252	148	52	193	215	198	240	177	184	233	149	64
124	169	162	171	157	143	227	238	145	10	181	219	251	187	141	247
95	235	255	208	226	91	214	19	182	48	92	134	185	107	112	77
111	248	150	190	166	63	236	151	231	180	191	223	153	131	205	1

### Decryption process

In this section, the construction of the decryption process is explained in the following steps.

1. **Stage 3:**

- Start with the encrypted augmented image, separate it into 3 color channels: Red, Green and Blue.
- Convert the color channels into *N* $2\times 2$ matrices $I_{[A,Red,Fredkin,Sbox,2\times 2,Hill]}$,
  $$I_{[A,Green,Fredkin,Sbox,2\times 2,Hill]}$$ and
  $$I_{[A,Blue,Fredkin,Sbox,2\times 2,Hill]}$$.
- Inverse matrices of Hill cipher are generated giving $Key'_{HillCipherRed}$,
  $$Key'_{HillCipherGreen}$$ and $$Key'_{HillCipherBlue}$$.
- $I_{[A,1D,Red,Fredkin,Sbox]}$ goes through matrix multiplication with $Key'_{HillCipherRed}$ giving $I_{[A,Red,Fredkin,Sbox,2\times 2]}$.
- $I_{[A,1D,Green,Fredkin,Sbox]}$ goes through matrix multiplication with $Key'_{HillCipherGreen}$ giving $I_{[A,Green,Fredkin,Sbox,2\times 2]}$.
- $I_{[A,1D,Blue,Fredkin,Sbox]}$ goes through matrix multiplication with $Key'_{HillCipherBlue}$ giving $I_{[A,Blue,Fredkin,Sbox,2\times 2]}$.

2. **Stage 2:**

- The inverse of 3 S-boxes are generated, giving $Sbox'_{6Dred}$, $Sbox'_{6Dgreen}$ and $Sbox'_{6Dblue}$.
- $I_{[A,Red,Fredkin,Sbox,2\times 2]}$ is converted into 1D byte-stream and invoked with $Sbox'_{6Dred}$ giving $I_{[A,Red,Fredkin,1D]}$.
- $I_{[A,Green,Fredkin,Sbox,2\times 2]}$ is converted into 1D byte-stream and invoked with $Sbox'_{6Dgreen}$ giving $I_{[A,Green,Fredkin,1D]}$.
- $I_{[A,Blue,Fredkin,Sbox,2\times 2]}$ is converted into 1D byte-stream and invoked with $Sbox'_{6Dblue}$ giving $I_{[A,Blue,Fredkin,1D]}$.

3. **Stage 1:**

- $I_{[A,Red,Fredkin,1D]}$ is converted into a bit-stream and divided into 2 halves as inputs ***B*** and ***C*** for the Fredkin gate with $Key_{GaussRed}$ as input ***A*** giving $I_{[A,1D,Red]}$.
- $I_{[A,Green,Fredkin,1D]}$ is converted into a bit-stream and divided into 2 halves as inputs ***B*** and ***C*** for the Fredkin gate with $Key_{GaussGreen}$ as input ***A*** giving $I_{[A,1D,Green]}$.
- $I_{[A,Blue,Fredkin,1D]}$ is converted into a bit-stream and divided into 2 halves as inputs ***B*** and ***C*** for the Fredkin gate with $Key_{GaussBlue}$ as input ***A*** giving $I_{[A,1D,Blue]}$.
- Finally, combine the 3 color channels into a single one and reshape back into an image forming the decrypted augmented image, which can split into multiple separate decrypted images.

**Fig. 3:**
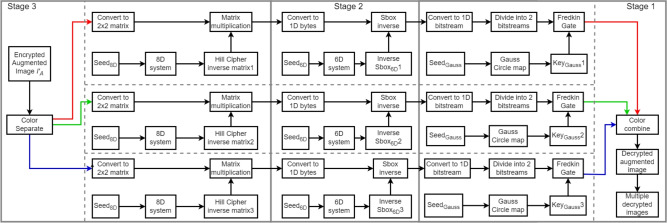
Decryption process flow chart.

An illustration of the decryption process is depicted in Fig. 3.

## Performance evaluation

This section conducts an extensive analysis using a computer that is equipped with an Intel^®^ Core^TM^ i7-7500U CPU at 2.7 GHz and 8 GB of RAM. Unless specified otherwise, the images used are enhanced and resized to $256\times 256$ pixels. Test images are sourced from 2 online collections: the USC-SIPI database^46^ and the MAR20 database^47^. Performance evaluation metrics commonly used in the image encryption scientific community are utilized in this article. These are presented in Table 5 and serve to quantitatively assess the performance of the proposed encryption scheme. The Peak Signal-to-Noise Ratio (PSNR) measures the similarity between the original and encrypted images. In encryption, a low PSNR value is desirable, as it indicates that the encrypted image is significantly different from the original, ensuring strong obfuscation. The Mean Squared Error (MSE) and Mean Absolute Error (MAE) quantify the average error between image pixels before and after encryption or decryption. Entropy evaluates the randomness of the encrypted image (with values close to 8 indicating high unpredictability, which is ideal for secure encryption). The Structural Similarity Index Measure (SSIM) assesses the structural similarity between original and decrypted images; a value of 1 confirms perfect reconstruction. The Number of Pixels Change Rate (NPCR) and the Unified Average Changing Intensity (UACI) measure the encryption’s sensitivity to small changes in the input, representing the algorithm’s robustness against differential attacks. The Discrete Fourier Transform (DFT) and the Pearson Correlation Coefficient (PCC) provide frequency and pixel correlation analyses, respectively, helping to evaluate how well the encryption disrupts inherent image patterns.

**Table 5:** Mathematical expressions of the performance evaluation metrics.

Metric	Descriptive equation(s)
SSIM	$SSIM(f,g) = l(f,g)^\alpha \cdot c(f,g)^\beta \cdot s(f,g)^\gamma$ where $l(f,g) = \frac{2\mu _x\mu _y + C_1}{\mu _x^2 + \mu _y^2 + C_1},$ $c(f,g) = \frac{2\sigma _x\sigma _y + C_2}{\sigma _x^2 + \sigma _y^2 + C_2},$ $s(f,g) = \frac{\sigma _{xy} + C_3}{\sigma _x \sigma _y + C_3}.$
MSE	$MSE = \frac{\sum _{i=0}^{M-1} \sum _{j=0}^{N-1} (I_{i,j} - I'_{i,j})^2}{M \times N}$
PSNR	$PSNR = 10 \log _{10} \left( \frac{I_{max}^2}{MSE}\right) , \quad I_{max} = 255$
MAE	$MAE = \frac{1}{M \times N} \sum _{i=0}^{M-1} \sum _{j=0}^{N-1} |P_{i,j} - E_{i,j}|$
Entropy	$H(m) = \sum _{i=1}^M p(m_i) \log _2 \frac{1}{p(m_i)}$
DFT	$F(k,l) = \sum _{i=0}^{N-1} \sum _{j=0}^{N-1} f(i,j) e^{-i 2\pi \left( \frac{ki}{N} + \frac{lj}{N}\right) }$
CC	$\rho (x,y) = \frac{\text {cov}(x,y)}{\sqrt{\sigma (x)} \sqrt{\sigma (y)}}, \quad \text {cov}(x,y) = \frac{1}{N} \sum _{i=1}^N (x_i - \mu _x)(y_i - \mu _y)$
NPCR	$NPCR = \frac{\sum _{x=1}^M \sum _{y=1}^N D(x,y)}{M \times N} \times 100,$ where $$D(x,y) = {\left\{ \begin{array}{ll} 0, & I(x,y) = I'(x,y) \\ 1, & \text {otherwise} \end{array}\right. }$$
UACI	$UACI = \frac{1}{M \times N} \sum _{x=1}^M \sum _{y=1}^N \frac{|I_1(x,y) - I_2(x,y)|}{255} \times 100$

### Visual and histogram analyses

**Fig. 4:**
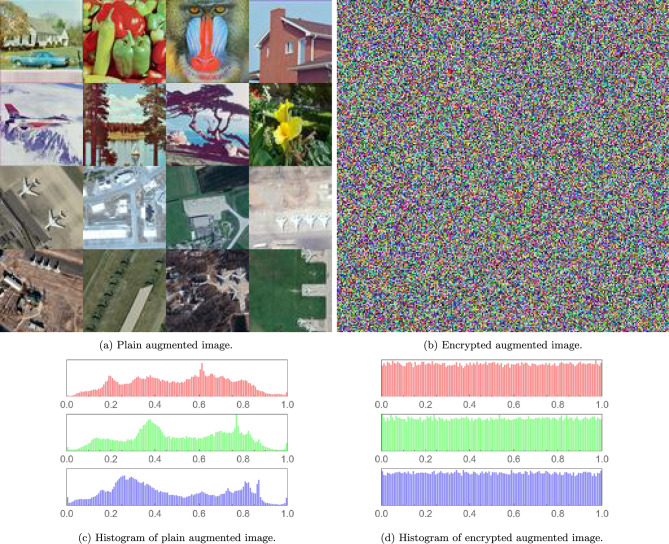
Images and histograms.

Figure 4 illustrates the transformation of a plain augmented image (a) into an encrypted noise-like augmented image (b) using the proposed encryption scheme. The histogram of the plain augmented image (c) exhibita distinct patterns in the RGB channels, reflecting the original content, while the histograms of the encrypted augmented image (d) shows uniform distributions, indicating successful diffusion and confusion. This uniformity ensures the obfuscation of all visual and statistical features, protecting the augmented data from cryptographic attacks. The results confirm the algorithm’s robustness in securing sensitive augmented images while preserving the integrity of the data format.

### Statistical analyses

The results presented in Table 6 demonstrate the efficacy of the proposed image encryption algorithm through various statistical metrics. The SSIM is consistently 1 for all test images, indicating perfect similarity between the decrypted and the original plain images, ensuring no structural distortion during the decryption process. However, the high MSE values and low PSNR values reflect significant encryption-induced transformations, which are essential for security. The MAE values further confirm notable pixel-level alterations. Additionally, the entropy metrics ($H_P$ and $H_E$) indicate high randomness in the encrypted images, with $H_E$ values close to the ideal upper bound of 8, affirming strong encryption quality. These metrics collectively validate the robustness and security of the proposed algorithm.

**Table 6:** Statistical analysis metrics of the proposed scheme in terms of various statistical metrics.

Image	SSIM	MSE	PSNR [dB]	MAE	$H_P$	$H_E$
Satellite Image 1	1	7195.21	9.56037	70.7714	7.31825	7.99891
Satellite Image 2	1	9819.98	8.2097	81.0019	7.19038	7.99878
Satellite Image 3	1	10332.0	7.98898	83.064	7.25253	7.9989
Mandrill	1	8307.08	8.93632	75.1588	7.69625	7.99895
Peppers	1	10061.6	8.10412	81.9657	7.71189	7.99869

Table 7 compares the Pearson Correlation Coefficient (PCC) values of plain and encrypted images across three directions: horizontal (H), diagonal (D), and vertical (V). The plain images exhibit high PCC values (averaging 0.91, 0.86, and 0.89 for H, D, and V, respectively), indicating strong pixel correlation and redundancy typical of natural images. In contrast, the encrypted images show near-zero PCC values (averaging $-0.00396$, $-0.001675$, and 0.000874 for H, D, and V, respectively), demonstrating that the proposed encryption algorithm effectively randomizes pixel relationships and eliminates structural dependencies. This highlights the algorithm’s robustness in ensuring image security by disrupting inherent correlations in the original images.

**Table 7:** PCC values computed for plain and encrypted images in three directions: horizontal (H), diagonal (D), and vertical (V).

Image	Plain Images (PCC)			Encrypted Images (PCC)		
	H	D	V	H	D	V
Satellite Image 1	0.888321	0.904528	0.904423	$-0.005642$	$-0.005415$	0.003279
Satellite Image 2	0.929822	0.84726	0.898305	0.000495	0.00331	$-0.0006066$
Satellite Image 3	0.941106	0.876438	0.928785	$-0.005468$	0.002015	0.006614
Mandrill	0.848778	0.750624	0.79088	$-0.003545$	$-0.004939$	0.001899
Peppers	0.959422	0.930426	0.966795	$-0.00564$	$-0.003348$	$-0.00681$
Average	0.9134898	0.8618552	0.8978376	$-0.003960$	$-0.001675$	0.000874

### Differential attacks analysis

Differential attacks evaluate an encryption algorithm’s sensitivity to slight changes in plaintext, with strong algorithms producing significantly different ciphertexts for minimal input changes. Metrics like NPCR (Number of Pixels Change Rate) and UACI (Unified Average Changing Intensity) assess this sensitivity. High NPCR values indicate significant pixel changes, while UACI measures the average intensity of those changes. As shown in Tables 8 and 9, the proposed algorithm achieves an average NPCR of 99.6115 and UACI of 31.70622, demonstrating robust resistance to differential attacks and consistent performance across diverse images, including benchmarks like “Mandrill” and “Peppers.” The slight deviation of the UACI value from the theoretical ideal of $33.46\%$ can be attributed to the structured nature of the augmented image and the deterministic components of the encryption process. While the method employs multiple chaotic layers and logical operations to enhance diffusion, components such as the structured Hill cipher and the need to preserve reversibility may slightly reduce pixel intensity variation. Nonetheless, the achieved UACI still reflects strong diffusion and confirms the robustness of the proposed scheme.

**Table 8:** NPCR values of encrypted images.

Image	Proposed
Satellite Image 1	99.6038
Satellite Image 2	99.6129
Satellite Image 3	99.6338
Mandrill	99.5824
Peppers	99.6246
Average	99.6115

**Table 9:** UACI values of encrypted images.

Image	Proposed
Satellite Image 1	32.5741
Satellite Image 2	31.7655
Satellite Image 3	32.5741
Mandrill	29.474
Peppers	32.1434
Average	31.70622

### Discrete fourier transform analysis

**Fig. 5:**
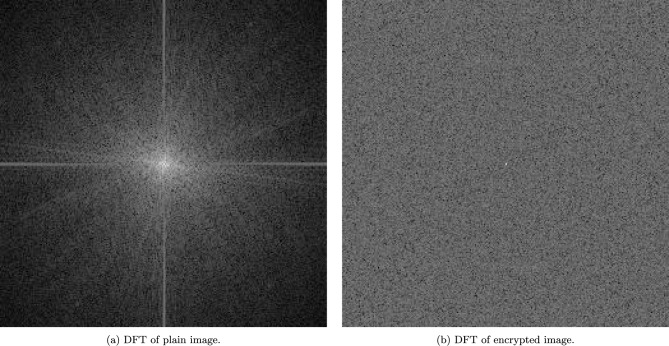
DFT as applied to plain and encrypted augmented images.

Figure 5 illustrates the Discrete Fourier Transform (DFT) applied to both a plain and an encrypted augmented image. The DFT of the plain image (a) shows a distinct concentration of frequency components at the center, indicating a significant amount of low-frequency information typical of unencrypted images. In contrast, the DFT of the encrypted image (b) presents a uniform distribution of frequency components, suggesting effective diffusion and obscuration of the original image’s frequency characteristics. This uniformity is indicative of high entropy and randomness in the encrypted image, which are desirable properties in secure image encryption schemes, demonstrating the effectiveness of the proposed encryption algorithm in dispersing the spectral components and enhancing the security of satellite images.

### Encryption time analyses

Table 10 presents the encryption time for augmented images of various dimensions, demonstrating the computational efficiency of the proposed algorithm. As the image size increases, the encryption time grows proportionally, ranging from 0.0727s for a $128\times 128$ image to 4.2098s for a $1024 \times 1024$ image. These results highlight the scalability of the method, making it suitable for encrypting high-resolution satellite images while maintaining practical processing times.

**Table 10:** Encryption time for various image dimensions.

Image dimensions	Time [s]
$128 \times 128$	0.0726797
$256 \times 256$	0.281687
$512 \times 512$	1.0182
$1024 \times 1024$	4.20981

### Big O complexity

The computational complexity of the proposed algorithm is ${\mathcal {O}}(M \times N)$, where *M* and *N* denote the dimensions of the input image. Table 11 presents a comparative analysis of the computational complexity of the proposed method with recent literature. The results indicate a comparable or superior performance to algorithms presented in recent literature.

**Table 11:** Computational complexity in terms of the big O notation.

Scheme	Time complexity
Proposed	$O(M\times N)$
^7^	$O(M\times N)$
^48^	$O(8\times M\times N\times L)$
^49^	$O(M\times N)$

### S-box performance analysis

S-boxes are critical in image encryption, providing non-linearity and resistance to attacks like differential and linear cryptanalysis. Table 12 shows the proposed S-boxes exhibit strong cryptographic properties, with nonlinearity (NL) and bit independence criterion (BIC) values (108) close to the ideal (112), SAC ($\approx 0.497$) near 0.5, and DAP matching the ideal (0.015625). These metrics confirm the S-boxes’ robustness and contribution to secure, efficient encryption.

**Table 12:** S-box evaluation metrics of the proposed algorithm.

S-box	NL	SAC	BIC	LAP	DAP
Prop. *S*1	108	0.49731	108	0.07813	0.015625
Prop. *S*2	108	0.49756	108	0.07813	0.015625
Prop. *S*3	108	0.49609	108	0.0781	0.015625
Ideal values	112	0.5	112	0.0625	0.015625

### Occlusion and noise attack analyses

**Fig. 6:**
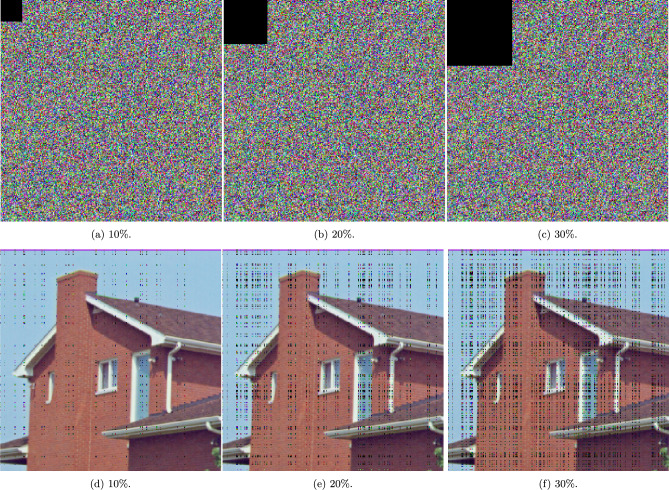
A visual representation of occlusion attacks with different percentages of occluded areas.

**Fig. 7:**
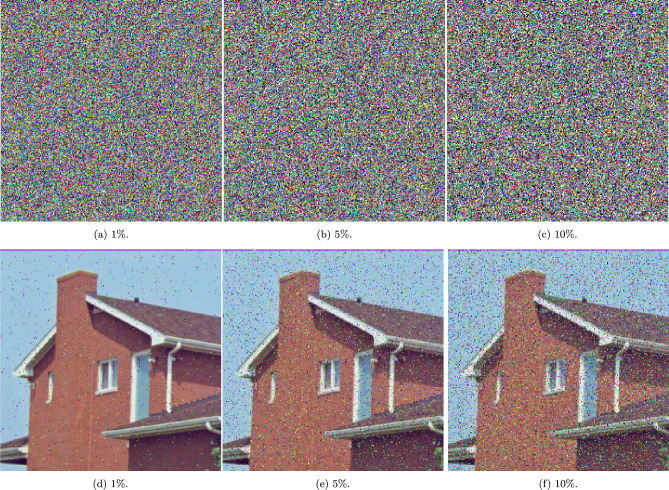
A visual representation of salt and pepper attacks with different intensities.

**Fig. 8:**
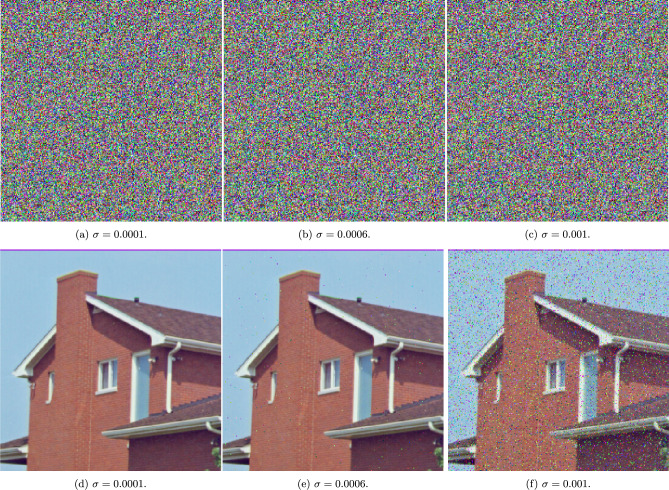
A visual representation of Gaussian noise attacks with different values of the noise standard deviation.

This section examines the resilience of the proposed encryption algorithm under various adverse conditions as depicted in Fig. 6, Fig. 7, and Fig. 8.

Fig. 6 focuses on the encryption’s performance against occlusion attacks, where black squares of varying sizes ($10\%$, $20\%$, and $30\%$ of the image area) simulate blocked portions of the image. The analysis shows that despite substantial occlusion, the encrypted image’s content remains secure and visually obscured, demonstrating the algorithms’s effectiveness in preserving data confidentiality even when significant parts of the image are obstructed. Furthermore, Table 13 shows the numerical analysis of various occlusion attack results in various images in comparison with the literature. The results indicate that the proposed encryption scheme results in higher MSE and lower PSNR under occlusion compared to existing methods, reflecting stronger security but lower robustness. While this reduces image recoverability and favors confidentiality, it highlights a trade-off with resilience to partial data loss.

Fig. 7 evaluates the scheme’s robustness against salt and pepper noise at different intensities ($1\%$, $5\%$, and $10\%$). The results indicate that the encrypted images maintain a high level of disorder, with no visible patterns or leakage of information, thereby underscoring the encryption’s capability to withstand disruptions caused by such noise.

Fig. 8 presents the impact of Gaussian noise with standard deviations $\sigma = 0.0001$, 0.0006, and 0.001 on the encrypted images. The images display a consistent preservation of visual integrity across increasing levels of noise, highlighting the scheme’s strong defense against statistical noise, which is typical in real-world scenarios involving signal transmission and storage.

Together, these figures effectively demonstrate the proposed algorithms’s substantial defensive properties against a range of occlusion and noise attacks, affirming its suitability for high-security satellite imaging applications where maintaining data integrity and confidentiality is paramount.

**Table 13:** Occlusion attack analysis on cropping sizes of 1/4 and 1/2.

Image	Scheme	1/4		1/2	
		MSE	PSNR [dB]	MSE	PSNR [dB]
Peppers	Proposed	11545.7	7.5066	10719.5	7.82905
	^50^	6570.4	10.5366	4856.1	12.1514
Airplane	Proposed	15045.4	6.35675	14495.8	6.51838
	^50^	6721.35	10.4501	4881.1	12.1164
Mandrill	Proposed	15045.4	6.35675	10819.4	7.78877
	^50^	6508.43	10.4249	478.21	12.5582
Lena	Proposed	12182.5	7.27343	11709.3	7.44551
	^50^	6478.29	11.0719	5003.19	13.0017
	^51^	1964.05	15.20	3908.68	12.21
House	Proposed	11686.0	7.45413	11519.3	7.51655
	^50^	5981.21	11.3127	4298.38	12.6317

### Key sensitivity analysis

**Fig. 9:**
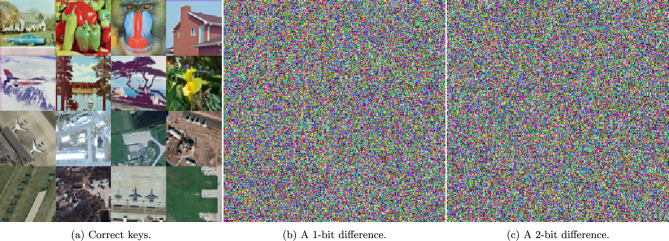
A visual representation of the key sensitivity by decrypting images using the correct keys, a key with a difference in a single bit, or two bits.

To illustrate the key sensitivity of the proposed algorithm, Fig. 9 displays various decryption scenarios. The first scenario demonstrates successful decryption using the correct keys, resulting in a perfectly reconstructed image. In contrast, changing a single bit or two bits in the decryption keys leads to failed decryption, where the recovered image becomes corrupted and loses its original information. This highlights the sensitivity of the decryption process to minor key modifications.

### NIST SP $800-22$ analysis

Table 14 summarizes results from the NIST suite of tests, which evaluate the randomness of sequences crucial for cryptographic security. Each test assesses different randomness aspects, with *p*-values indicating the likelihood that a sequence is random. A *p*-value greater than 0.01 typically suggests acceptable randomness. The results consistently marked as ’Success’ across various tests, including Frequency, Block Frequency, Runs, Serial, Random Excursions, and their Variants, confirm the robust randomness of the tested sequences. This demonstrates the effectiveness of the encryption method in producing secure, random sequences essential for reliable cryptographic applications.

**Table 14:** Results of the NIST suite of tests.

Test	*p*-value	Conclusion
Frequency	0.593182	Success
Block Frequency	0.477881	Success
Run	0.595548	Success
Long runs of ones	0.623060	Success
Rank	0.622479	Success
Spectral F.F.T.	0.325403	Success
Non overlapping	0.963474	Success
Overlapping	0.662890	Success
Universal	0.474979	Success
Serial	0.252341	Success
Serial	0.319291	Success
Approx. entropy	0.797941	Success
Cum. sums forward	0.960674	Success
Cum. sums reverse	0.520303	Success
R.E. 1	0.908986	Success
R.E. 2	0.920043	Success
R.E. 3	0.944351	Success
R.E. 4	0.292942	Success
R.E. 5	0.130057	Success
R.E. 6	0.044350	Success
R.E. 7	0.115114	Success
R.E. 8	0.121972	Success
R.E.V. 1	0.971018	Success
R.E.V. 2	0.827767	Success
R.E.V. 3	0.432919	Success
R.E.V. 4	0.384596	Success
R.E.V. 5	0.648645	Success
R.E.V. 6	0.904232	Success
R.E.V. 7	0.603624	Success
R.E.V. 8	0.488999	Success
R.E.V. 9	0.985060	Success
R.E.V. 10	0.245659	Success
R.E.V. 11	0.242974	Success
R.E.V. 12	0.769450	Success
R.E.V. 13	0.793425	Success
R.E.V. 14	0.591414	Success
R.E.V. 15	0.530863	Success
R.E.V. 16	0.585541	Success
R.E.V. 17	0.642546	Success
R.E.V. 18	0.873706	Success

### Key space analysis

Key space is a crucial measure for assessing the security of an encryption scheme against brute force attacks. The system is constructed from the hyperchaotic system in (3) which utilizes 15 parameters for each single color channel. In addition, the S-boxes generated come from the hyperchaotic system in (2) which utilizes 12 parameters for each color channel. Finally, the Gaussian map in (1) utilizes 5 parameters that generate a bit-stream for every color channel that is utilized in the Fredkin gate operation, where the total will be $15\times 3 + 12\times 3+5\times 3=96$ and with a maximum machine precision of $10^{-16}$, the key space is $10^{16\times 96}=10^{1536}$, which is approximately $2^{5102}$. This achieved key space is well above the literature-recommended threshold of $2^{100}$^52^.

### Comparative analysis with the literature

A comparative analysis is shown in Table 15, where the performance of various image encryption algorithms is displayed, including the proposed one that is tailored specifically for satellite images. In particular, the proposed algorithm achieves a lower PSNR and a higher MAE compared to other methods, which is advantageous in this context as it suggests stronger encryption (lower PSNR indicates less similarity between the original and encrypted images, and higher MAE indicates more significant alterations). Entropy values close to the maximum and very low or negative correlation coefficients across the proposed method indicate robust randomness and minimal resemblance to the original, enhancing security against statistical attacks. Additionally, the high NPCR values and reasonable UACI values further confirm the sensitivity and intensity of the modifications, underscoring the effectiveness of the proposed method in securing sensitive geographic data through advanced encryption techniques. This analysis highlights the suitability of the proposed method for high-security applications such as satellite imagery, where data integrity and confidentiality are paramount.

**Table 15:** Comparative analysis of various algorithms from the literature.

Image		PSNR	MAE	Entropy	CC			NPCR	UACI
					H	D	V		
Peppers	Proposed	8.10412	81.9657	7.99869	$-0.0056417$	$-0.00334876$	$-0.0068176$	99.6246	32.1434
	^20^	8.12503	81.718	-	0.00430376	$-0.00544425$	$-0.00181753$	-	-
	^27^	39.220	-	-	-	-	-	99.787	33.621
	^28^	9.55	-	-	$-0.0028$	$-0.0090$	$-0.0007$	99.5626	33.5486
	^29^	-	-	7.9971	0.0021	0.0001	0.0002	100	33.4861
	^30^	8.13789	82.17790	7.99916	$-0.00021$	0.00027	0.00128	99.60501	32.22662
	^31^	-	-	-	0.0008	0.0013	0.0011	-	-
	^37^	8.09689	82.0274	7.99908	0.00580309	0.00245556	$-0.00265354$	99.6272	32.1676
Mandrill	Proposed	8.93632	75.1588	7.99895	$-0.00354504$	$-0.00493905$	0.00189976	99.5824	29.474
	^20^	8.92043	75.2984	-	0.00509484	0.00356122	$-0.00101199$	-	-
	^27^	39.134	-	-	-	-	-	99.881	33.415
	^28^	10.10	-	-	$-0.0029$	$-0.0079$	0.0026	99.5926	33.4496
	^29^	-	-	7.997	0.003	-	0.0002	100	33.7145
	^30^	8.9272	75.49169	7.99865	$-0.00004$	$-0.00021$	0.00039	99.60480	29.60459
	^31^	7.7447	92	7.9991	0.0011	$-0.0001$	0.0003	99.61	33.51
	^37^	8.93946	75.1327	7.99914	$-0.00298955$	$-0.00309558$	$-0.0000746902$	99.6185	29.4638

The results in Table 16 highlight the superior computational efficiency of the proposed scheme, with an encryption time of 0.2817s, significantly faster than 0.7528s in^20^ and 3.0019s in^30^. All tests were conducted on relatively comparable hardware, demonstrating the practicality of the proposed algorithm for real-time satellite image encryption while maintaining robust security.

**Table 16:** Encryption time comparison with counterpart algorithms from the literature.

Scheme	Time [s]	Machine Specifications
Proposed	0.281687	AMD^®^ Ryzen^TM^ 5600H, 3.3 GHz, 16 GB
^20^	0.752824	AMD^®^ Ryzen^TM^ 5600H Mobile 3.3 GHz, 16 GB
^21^	0.44	3.3 GHz AMD^®^ Ryzen 9 5900HX, 32 GB
^30^	3.0019	3.4 GHz Intel^®^ Core^TM^ i7, 8 GB
^36^	0.162179	3.3 GHz AMD^®^ Ryzen 9 5900HX, 32 GB
^37^	0.72	N/A

Table 17 shows that the proposed S-boxes demonstrate strong overall performance compared to those in the literature, with excellent results in Differential Approximation Probability (DAP), matching the ideal value of 0.015625, and competitive Linear Approximation Probability (LAP) values (0.07813), outperforming many referenced S-boxes. While the Non-Linearity (NL) values (108) are slightly below the ideal (112) and some top-performing S-boxes, they remain competitive. The Strict Avalanche Criterion (SAC) values $(\approx 0.497)$ are close to the ideal (0.5), and the Bit Independence Criterion (BIC) values (108) are consistent and comparable to most counterparts. Overall, the proposed S-boxes strike a strong balance between cryptographic strength and practical performance, making them viable for secure image encryption applications, with room for minor improvements in NL and SAC.

**Table 17:** S-box evaluation metrics of various algorithms from the literature.

S-box	NL	SAC	BIC	LAP	DAP
Proposed (average)	108	0.496989	108	0.07813	0.015625
^20^	110	0.5056	108	0.0781	0.0156
^31^	106	0.497	96	0.125	0.015625
^32^	100	0.5007	104.1	0.390	0.1250
^33^	111	0.5036	110	0.0781	0.0234
^34^	103.5	0.5065	103.3	0.1328	0.0468
^35^	107	0.497	103.5	0.1560	0.0390
Ideal values	112	0.5	112	0.0625	0.015625

The key space of an encryption algorithm is a critical factor in determining its resistance to brute-force attacks. Table 18 compares the key space of various image encryption algorithms from the literature, highlighting the significant advantage of the proposed method. With a key space of $2^{5102}$, the proposed algorithm surpasses most existing methods, including those with substantial key spaces such as^36^ $2^{4624}$ and^30^ ($2^{554}$). Notably, some prior works, such as^21^, have considerably smaller key spaces ($>2^{100}$), making them more vulnerable to exhaustive search attacks. The formulation $2^{8 \times M \times N}$ in^29^ suggests a dependency on image dimensions, which may offer scalability but also variability in security. Overall, the proposed algorithm’s exceptionally large key space provides a robust defense against brute-force attacks, reinforcing its suitability for secure image encryption.

**Table 18:** Key space of various algorithms from literature.

Algorithm	Key space
Proposed	$2^{5102}$
^21^	$>2^{100}$
^29^	$2^{8\times M \times N}$
^30^	$2^{554}$
^36^	$2^{4624}$
^37^	$2^{212}$

## Conclusions and future works

This work presented a multi-stage chaos-based encryption scheme designed specifically for satellite images. The proposed algorithm integrates classical cryptographic techniques with chaotic systems, including the Gauss Circle map, Fredkin gates, hyperchaotic key generation, and dynamic Hill Ciphers. By augmenting and encrypting multiple images simultaneously, the method enhances both security and processing efficiency.

The encryption process achieves high levels of confusion and diffusion, as evidenced by strong statistical metrics such as entropy values near the ideal of 8, NPCR rates exceeding $99.6\%$, and low PSNR values, all indicating robust resistance to differential and statistical attacks. Additionally, the scheme maintains computational efficiency, with an encryption time of 0.2817 seconds for $256 \times 256$ images, and demonstrates resilience to various attacks, including noise, occlusion, and brute-force attempts.

Despite its strengths, the proposed scheme has a few limitations. The image augmentation step increases memory usage, which may pose challenges for resource-constrained environments. The reliance on high-precision chaotic systems can complicate hardware implementation. Furthermore, while the algorithm performs well in software simulations, further validation on real-time hardware platforms is necessary to confirm its practical applicability in real-world satellite systems.

Future research will focus on addressing these limitations by exploring lightweight hardware implementations, adaptive parameter tuning, and optimizing the algorithm for deployment in constrained or real-time environments. Additionally, integrating the encryption scheme with secure transmission protocols and evaluating its performance on edge-processing satellite platforms are promising directions for further development.

## Data Availability

The datasets analyzed in this study are available from the corresponding author on reasonable request.
